# Supplementary material for: A novel mRNA-based multiepitope vaccine candidate against Cryptosporidium hominis and Cryptosporidium parvum employing reverse-vaccinology and immunoinformatics approaches
Source: PLoS One. 2026 Feb 25;21(2):e0343643. doi: 10.1371/journal.pone.0343643 (PMC12935263; doi:10.1371/journal.pone.0343643)
Supplement: S1 Table — (DOCX) [file pone.0343643.s003.docx]

**S1 Table.** The two-dimensional structures of the vaccine.

| Properties | GOR4 | SOPMA |
| --- | --- | --- |
| Alpha helix (Hh) | 225 (43.02%) | 197 (37.67%) |
| 3_10_ helix (Gg) | 0.00% | 0.00% |
| Pi helix (Ii) | 0.00% | 0.00% |
| Beta bridge (Bb) | 0.00% | 0.00% |
| Extended strand (Ee) | 54 (10.33%) | 66 (12.62%) |
| Beta turn (Tt) | 0.00% | 47 (7.57%) |
| Bend region (Ss) | 0.00% | 0.00% |
| Random coil (Cc) | 244 (46.65%) | 222 (42.45%) |
