# Supplementary material for: A novel mRNA-based multiepitope vaccine candidate against Cryptosporidium hominis and Cryptosporidium parvum employing reverse-vaccinology and immunoinformatics approaches
Source: PLoS One. 2026 Feb 25;21(2):e0343643. doi: 10.1371/journal.pone.0343643 (PMC12935263; doi:10.1371/journal.pone.0343643)
Supplement: S2 Table — (DOCX) [file pone.0343643.s004.docx]

**S2 Table.** The predicted discontinuous B-cell epitopes for vaccine.

| **No** | **Residues** | **Number of residues** | **Scores** |
| --- | --- | --- | --- |
| 1 | A:M1, A:A2, A:K3 | 3 | 0.993 |
| 2 | A:L4, A:S5, A:T6, A:D7, A:E8, A:L9, A:L10, A:D11, A:A12, A:F13, A:K14, A:E15, A:M16, A:T17, A:L18, A:L19, A:E20, A:L21, A:S22, A:D23, A:F24, A:V25, A:K26, A:K27, A:F28, A:E29, A:E30, A:T31, A:F32, A:V34, A:T35, A:A36, A:A37, A:A38, A:P39, A:V40, A:A41, A:V42, A:A43, A:A44, A:A45, A:G46, A:A47, A:A48, A:P49, A:A50, A:G51, A:A52 | 48 | 0.852 |
| 3 | A:E463, A:K464, A:F465, A:E466, A:R467, A:Y468, A:Q469, A:K471, A:D472, A:S473, A:N474, A:Q475, A:N478, A:S479, A:E480, A:K481, A:A482, A:P483, A:K484, A:F485, A:E486, A:R487, A:S488, A:G489, A:S490, A:A491, A:G492, A:T493, A:A494, A:T495, A:E496, A:S497, A:T498, A:A499, A:T500, A:T501, A:T502 | 37 | 0.849 |
| 4 | A:R447, A:E448, A:D449, A:E450, A:G451, A:E452, A:K454, A:E455, A:E456, A:G457, A:R458, A:K459, A:R460, A:R461, A:W462 | 15 | 0.757 |
| 5 | A:E360, A:K361, A:S362, A:E363, A:S364, A:H368, A:S376, A:S377, A:K378, A:S379, A:N382, A:K384, A:E386, A:R387, A:P388, A:H389, A:M390, A:S391, A:E392, A:Y393, A:T394, A:P395, A:D396, A:K397, A:Y398, A:R400, A:R401, A:F403, A:F405, A:E406, A:R407, A:A408, A:Q409, A:A410, A:P411, A:P412, A:A413, A:P414, A:A415, A:E416, A:P417, A:A418, A:P419, A:Q420, A:D421, A:K422, A:P423, A:K424, A:F425, A:E426, A:R427, A:Q428, A:K429, A:P430, A:E431, A:E432, A:P433, A:K434, A:K435, A:S436, A:E437, A:P438, A:A439, A:S440, A:N441, A:N442, A:P443, A:K444, A:F445 | 69 | 0.699 |
| 6 | A:L67, A:A69, A:A70, A:G71, A:D72, A:K73, A:K74, A:I75, A:G76, A:V77, A:I78, A:K79, A:V80, A:V81, A:R82, A:E83, A:V85, A:S86, A:G87, A:L88, A:G89, A:L90, A:K91, A:E92, A:A93, A:K94, A:D95, A:L96, A:V97, A:D98, A:G99, A:A100, A:P101, A:K102, A:A122, A:A123, A:G124, A:A125 | 38 | 0.68 |
| 7 | A:S515, A:G516, A:S517, A:A518, A:G519, A:T520, A:A521, A:T522, A:E523 | 9 | 0.646 |
| 8 | A:K165, A:K168, A:A169, A:P170, A:Q171, A:D172, A:K173, A:P174, A:A175, A:E176 | 10 | 0.57 |
| 9 | A:R227, A:E260, A:P272, A:P278, A:E279, A:E280, A:P281, A:K282, A:K283, A:S284, A:E285, A:P286, A:A287, A:S288, A:N289, A:N290, A:P291 | 17 | 0.557 |
| 10 | A:Y139, A:T140, A:P141, A:D142 | 4 | 0.53 |
| 11 | A:K264, A:S265, A:E266, A:P267, A:A268, A:S269, A:N270, A:N271, A:D339 | 9 | 0.528 |
