# Supplementary material for: A novel mRNA-based multiepitope vaccine candidate against Cryptosporidium hominis and Cryptosporidium parvum employing reverse-vaccinology and immunoinformatics approaches
Source: PLoS One. 2026 Feb 25;21(2):e0343643. doi: 10.1371/journal.pone.0343643 (PMC12935263; doi:10.1371/journal.pone.0343643)
Supplement: S3 Table — (DOCX) [file pone.0343643.s005.docx]

**S3 Table.** The energy scores of MM-GBSA for the complexes.

| Complex | VDW  (kcal/mol) | ELE  (kcal/mol) | GB  (kcal/mol) | SA  (kcal/mol) | Total binding energy  (kcal/mol) |
| --- | --- | --- | --- | --- | --- |
| Vaccine-TLR-2 | -125.07 | 528.6 | -451.22 | -15.61 | -63.3 |
| Vaccine-TLR-4 | -187.78 | 1082.03 | -940.48 | -23.94 | -70.17 |
