# Supplementary material for: A novel mRNA-based multiepitope vaccine candidate against Cryptosporidium hominis and Cryptosporidium parvum employing reverse-vaccinology and immunoinformatics approaches
Source: PLoS One. 2026 Feb 25;21(2):e0343643. doi: 10.1371/journal.pone.0343643 (PMC12935263; doi:10.1371/journal.pone.0343643)
Supplement: Attachment — Submitted filename: Response to Reviewers.docx [file pone.0343643.s006.docx]

**Response to Reviewers**

**Reviewer #1:**

Biological experiments should be conducted to develop the vaccine. This is a bioinformatic manuscript for vaccine development (PONE-D-25-20787). The language and writing are fine. However, this manuscript is not of great importance to developing the *Cryptosporidium parvum* vaccine.

**Response:** We respectfully recognize the reviewer's concern regarding the lack of *in-vitro* or *in-vivo* validation. As indicated in the manuscript, the current research is structured as an *in-silico* discovery and prioritization framework, constituting a fundamental step toward experimental vaccine development. Due to the significant expenses, labor demands, biosafety protocols, and ethical considerations involved in handling *Cryptosporidium parvum*, computational vaccine screening is extensively employed as an initial step to identify potential antigenic targets prior to laboratory validation. *In-silico* approaches have become recognized in modern vaccinology for:

1. Minimizing prospective failure rates prior to animal or human testing.

2. Reducing expenses and time through early exclusion of non-immunogenic targets.

3. Supporting researchers without direct access to pathogen culture facilities, especially for organisms subject to stringent biosafety protocols such as *Cryptosporidium parvum*.

We firmly believe that the predicted epitopes and validated antigenic constructs produced in this study offer a logical foundation for subsequent wet-lab assessment. Multiple mRNA and peptide vaccines, including SARS-CoV-2 immunogens, were initially developed employing comparable computational approaches prior to experimental validation.

**Reviewer #2:**

**1.** This is a well written manuscript, however some for the processes in the methodology could be further summarized.

**Response:** The methodology has been summarized now.

**2.** The study aim was well stated in lines 148-150 but “integrating vaccines” shouldn’t be part of the aim. It fits best in the introduction section.

**Response:** The line is revised as **“**The efficacy of mRNA vaccines and RV served as a substantial impetus for us, as both approaches facilitate the identification of ideal vaccine constituents and yield safe, stable and effective vaccines.”

**3.** It would be nice to structure the abstract

**Response:** The abstract has been structured now. Please, refer to the abstract section of the article.

**4.** The conclusion as written in the abstract is not appropriate it should be revised and stated in line with the study aim. While the Authors have tried to state the conclusion from the or study, they have also included some discussion of their conclusion. The conclusion should be revised and clearly stated in line with the study aim and leave out the discussion.

**Response:** The conclusion in the abstract has been updated as “This study developed an in silico multiepitope mRNA vaccine candidate for *Cryptosporidium hominis* and *Cryptosporidium parvum* with excellent structural stability, antigenicity, receptor-binding affinity, and expected immune responses. These findings offer a foundational concept for experimental validation.”

**Reviewer #3:**

The study presents a computational design of an mRNA-based multiepitope vaccine targeting *Cryptosporidium hominis* and *C. parvum.* While the topic is relevant and aligns with the current trend of using immuno-informatics for vaccine development, the manuscript lacks sufficient methodological rigor, biological validation, and critical interpretation of results. Several sections read like a procedural description rather than a scientific analysis, and there are substantial concerns regarding novelty, clarity, data reproducibility, and presentation. Hence, major revision is strongly recommended before this manuscript can be considered for publication. While the topic is timely and the approach relevant, the manuscript currently lacks methodological rigor, interpretative depth, and structural clarity. The claims of strong immunogenicity and universal coverage are unsupported by robust comparative analysis or experimental evidence. A comprehensive revision with clear validation, data justification, and scientific moderation is required. Major Issues That Must Be Addressed
1. Scientific validation: Add benchmarking or comparison to previously published vaccine constructs or experimental epitopes.

**Response:** The benchmarking or comparison to previously published vaccine constructs or experimental epitopes have been added now to the manuscript.

2. Novelty and contribution: Clarify what this study adds beyond existing in-silico works on Cryptosporidium.

**Response:** The novelty of this vaccine has been added in the discussion section as **“**As five of the vital proteins were targeted specifically along with dual species of parasites, this current study is novel and superior to the previously mentioned studies.”
3. Data reproducibility: Provide detailed parameters, thresholds, and URLs for all tools, including dataset accession IDs.

**Response:** The detailed parameters, thresholds, and URLs for all tools, including dataset accession IDs are added now.

4. Figures and tables: Improve quality, labeling, and consistency with captions and in-text references.

**Response:** The figure and table quality labeling, and consistency with captions and in-text references have been improved now.

5. Discussion: Critically interpret findings and discuss biological limitations of in-silico work.

**Response:** The findings have been critically interpreted and the biological limitations are added now in the discussion section.

6. Language: Substantial language polishing and reorganization needed to improve readability.

**Response:** The language has been polished now with the addition of fixed grammatical mistakes
7. Ethical and data statements: Ensure that the data availability and ethical statements follow journal standards and are placed at the end of the manuscript.

**Response:** The data availability statement was already there at the end of the manuscript however, the ethical declaration has been added at the end of the manuscript as “This research study does not use human or animal models. Therefore, no ethical statement is relevant to this investigation.”

My detailed Comments are given as follows

**1. Title and Abstract**

- The title is clear but overly long. Consider shortening to emphasize the main objective (e.g., “In-silico design of a multiepitope mRNA vaccine against Cryptosporidium spp. using reverse vaccinology”).

**Response:** The title has been shortened as “A novel mRNA-based multiepitope vaccine candidate against *Cryptosporidium hominis* and *Cryptosporidium parvum* employing Reverse-Vaccinology and Immuno-informatics approaches”

- The abstract is overly descriptive and lacks quantitative insight into why this vaccine design is superior or novel compared to prior in-silico vaccine models for Cryptosporidium.

**Response:** The abstract has been fixed now with quantitative insights, novelty and drawbacks as **“**These findings offer a novel approach due to numerous species target but with significant drawbacks like no validation beyond simulation, uncertainty of long-term immunity, protein quality, stability and safety, requiring experimental validation.**”**

- Statements such as “we firmly endorse ongoing research” are subjective and inappropriate for a scientific abstract.

**Response:** This statement has been removed now from the manuscript.

- No validation beyond computational simulation is performed; this limitation should be explicitly stated in the abstract.

**Response:** The limitation is mentioned now as **“**These findings offer a novel approach due to numerous species target but with significant drawbacks like no validation beyond simulation, uncertainty of long-term immunity, protein quality, stability and safety, requiring experimental validation.**”**

**2. Introduction**

- The introduction provides good epidemiological background but is excessively lengthy, with large portions of historical data that distract from the rationale.

**Response:** The epidemiological background is shortened now as “Cryptosporidiosis was initially identified in humans during the late 1970s, predominantly impacting immunocompromised individuals, with a significant rise in cases noted at the onset of the AIDS epidemic in the early 1980s. Since 2005, reported cases in the United States have increased significantly, partly attributable to the emergence of the *Cryptosporidium hominis* subtype IaA28R4. Surveillance data reveal significant fluctuations in incidence, with marked rises in outbreaks and related hospitalizations across several states, especially in the Great Lakes region. Beyond human health, cryptosporidiosis continues to be a significant issue in veterinary medicine, with bovine infections representing a primary source of calf enteritis globally, thereby exerting substantial economic pressures on livestock production systems.”

- The research gap is not sharply defined. The authors should clearly state what previous in-silico vaccine studies exist for Cryptosporidium and what new contribution this work makes (e.g., targeting both *C. hominis* and *C. parvum* together).

**Response:** The research gap, previous vaccine studies are mentioned now in the manuscript.

- Many statements lack references to recent literature (e.g., 2023-2025 studies on mRNA vaccine modeling or reverse vaccinology). The following articles are worthy to read and cite.
- https://doi.org/10.15586/qas.v15i1.1210
- https://doi.org/10.1016/j.intimp.2024.113345
- https://doi.org/10.1038/s41598-025-98151-4
- https://doi.org/10.1038/s41598-025-96989-2
- https://doi.org/10.1080/19476337.2023.2296006
- https://doi.org/10.1038/s41598-025-00166-4
- https://doi.org/10.1007/s12033-025-01477-7
- https://doi.org/10.1016/j.intimp.2024.113241
- https://doi.org/10.1016/j.intimp.2025.115492
- <https://doi.org/10.31083/j.fbl2905176>

**Response:** We have gone through the mentioned articles, inputted information where necessary and properly cited the appropriate articles that aligns with our study.

- The transition from epidemiology to computational vaccine design is abrupt. The hypothesis and study objectives should be concisely stated.

**Response:** The study objective has been fixed as “Given the need, this study focuses on designing a potential vaccine candidate to overcome the effects of cryptosporidiosis.”

**3. Materials and Methods**

This section is highly procedural and lists tools/servers without methodological justification or parameter explanation.

**Response:** The methodological justification or parameter explanation have been added now.

**Critical missing details:**

Threshold values used for epitope screening (e.g., VaxiJen score cutoffs).

Criteria for selecting final epitopes among predicted candidates.
Validation of selected proteins’ antigenicity and conservation.
Details on population coverage computation and specific HLA alleles used.
Lack of description of how structural models were verified for quality (no mention of validation metrics besides RMSD and Ramachandran).

**Response:** The criteria for selecting final epitopes among predicted candidates, validation of selected proteins’ antigenicity, population coverage and structural verification have been improved now on the manuscript.

No information is provided on control or benchmarking against known vaccine epitopes.

**Response:** For *in-silico* studies, controls are not applied in the same way, as these rely on computer simulations and predictive algorithms to assess the construct’s potential without physical comparisons [1-4].

• There is no statistical analysis or error quantification — all outputs are descriptive.

**Response:** In *in-silico* studies, statistical tests are not conducted because, these are solely based on algorithms and software that provide result in by default implemented statistical tests. In similar type of studies, no statistical tests are not conducted [1-4].

**References**

1.	Fathollahi M, Fathollahi A, Motamedi H, Moradi J, Alvandi A, Abiri R. In silico vaccine design and epitope mapping of New Delhi metallo-beta-lactamase (NDM): an immunoinformatics approach. BMC bioinformatics. 2021;22(1):458. doi: 10.1186/s12859-021-04378-z.

2.	Saha S, Vashishtha S, Kundu B, Ghosh M. In-silico design of an immunoinformatics based multi-epitope vaccine against Leishmania donovani. BMC bioinformatics. 2022;23(1):319. doi: 10.1186/s12859-022-04816-6.

3.	Yang Z, Bogdan P, Nazarian S. An in silico deep learning approach to multi-epitope vaccine design: a SARS-CoV-2 case study. Scientific reports. 2021;11(1):3238. doi: 10.1038/s41598-021-81749-9.

4.	Evangelista FMD, van Vliet AHM, Lawton SP, Betson M. In silico design of a polypeptide as a vaccine candidate against ascariasis. Scientific reports. 2023;13(1):3504. doi: 10.1038/s41598-023-30445-x.

• The pipeline lacks reproducibility — for instance, figures and tables appear referenced but are not accompanied by clear legends or supporting data.
**Response:** The data reproducibility has been improved now.

**4. Results**

The results are purely descriptive, with minimal interpretation.

Most figures and tables are referred to but not critically discussed.
• The claim of “100% global population coverage” is highly unrealistic and likely reflects overfitting due to inclusion of all alleles — needs recalculation and justification.

**Response:** The claim of “100% global population coverage” probably stems from the incorporation of an excessively broad array of HLA alleles, which can falsely enhance coverage estimates in computer analysis. Predictions of population coverage are essentially contingent upon the chosen HLA alleles, binding thresholds, and the comprehensiveness of existing allele-frequency databases; thus, they should be regarded as probabilistic rather than definitive metrics. To alleviate this concern, population coverage must be recalculated exclusively with the specific HLA class I and II alleles to which the selected epitopes are demonstrably bound under established affinity thresholds, and results should be presented for global and regional populations distinctly. The interpretation is changed to show high predicted population coverage instead of full global coverage.

• The docking and simulation results are not compared with any known control peptides or standard vaccines, making their biological significance unclear.

**Response:** The docking and simulation results are now compared with previous studies now not controls as the in-silico studies are solely on algorithm based.
• The MM-GBSA results are reported without context; the energy values alone cannot confirm strong or weak binding without benchmarking.

**Response:** The MM-GBSA results are now reported with benchmarks on the discussion section.

The immune simulation results (C-ImmSim) lack comparative baseline; hence, the conclusions on immune protection are speculative.

**Response:** The comparative baseline is now added to the discussion section for the immune simulation

Figures lack sufficient resolution and labels; color coding (e.g., for linkers and epitopes) is not standardized or scientifically meaningful.

**Response:** The vaccine construction figure is updated with standard colors and high resolution. Additionally, the other figures were checked and high quality was achieved.
**5. Discussion**

The discussion mostly repeats results and does not critically analyze limitations or uncertainties in computational predictions. There is overinterpretation of in-silico results as if they were experimentally validated. No comparison is made with existing in-silico vaccine designs for other protozoan parasites (e.g., Toxoplasma, Giardia), which could provide important context.
• Statements about global applicability, long-term immunity, and biological stability are speculative without in-vitro or in-vivo validation.

**Response:** The discussion is fully modified with critically analyzing limitations of computational prediction, comparison with previous studies.

**6. Conclusion**

- The conclusion is overly optimistic and unbalanced — it should acknowledge the limitations of computational predictions and the need for experimental verification.

**Response:** The limitations of computational predictions are mentioned now on the manuscript as “Nevertheless, this study focused solely on computational prediction, which has some limitations, including a lack of long-term immunity prediction, structural prediction errors, and uncertainty about adjuvant interactions, and thus requires preclinical and clinical trials for validation.”

- The statement “the vaccine was predicted to be marginal in stimulating immune response” contradicts earlier claims of strong immunogenicity — this needs clarification

**Response:** The statement has been fixed now as “The vaccine was potential in activating various immune cells (T and B cells), cytokines and antibodies.”

**7. Language and Formatting**

- The manuscript contains numerous grammatical issues, redundant phrases, and inconsistent use of scientific terms.

**Response:** All the grammatical issues, redundant phrases, and inconsistent use of scientific terms have been fixed now.

- Citation formatting is inconsistent (e.g., missing brackets, outdated numbering).

**Response:** The citation formatting has been fixed now.

- Figures and tables are not clearly referenced or formatted according to PLOS ONE style.

**Response:** The figures and tables are now clearly referenced or formatted according to PLOS ONE style.

- The tone should be scientific and neutral remove promotional or conclusive phrases like “firmly endorse ongoing research.”

**Response:** All the tones and phrases have been checked and promotional or conclusive phrases have been removed.
