# Supplementary material for: A novel mRNA-based multiepitope vaccine candidate against Cryptosporidium hominis and Cryptosporidium parvum employing reverse-vaccinology and immunoinformatics approaches
Source: PLoS One. 2026 Feb 25;21(2):e0343643. doi: 10.1371/journal.pone.0343643 (PMC12935263; doi:10.1371/journal.pone.0343643)
Supplement: Attachment — Submitted filename: REVIEWERS COMMENTS.docx [file pone.0343643.s007.docx]

REVIEWERS COMMENTS

In the Revised Manuscript the authors have adequately responded to the reviewer’s comments and recommendation. The manuscript can be accepted for publication if the journal requirements have been fulfilled.
